# Early Reconstruction with Locoregional-Free Flaps in Post-COVID-19 Rhino-orbital-cerebral Mucormycosis Craniofacial Deformities: A Single-Center Clinical Experience from India

**DOI:** 10.1055/s-0043-1778652

**Published:** 2024-01-15

**Authors:** Veena K. Singh, Ansarul Haq, Sarsij Sharma, Anupama Kumari

**Affiliations:** 1 Department of Burns & Plastic Surgery, All India Institute of Medical Sciences, Patna, Bihar, India

**Keywords:** COVID-19, mucormycosis, reconstruction, facial

## Abstract

**Aim of the Study** Mucormycosis is a rare invasive and fatal fungal infection and its resurgence in coronavirus disease 2019 (COVID-19) patients has been a matter of grave concern. It is essentially a medical disease, but surgical debridement of necrotic tissues is of paramount importance leading to severe craniofacial deformities. In this case series, we present our experience with the feasibility of early reconstruction after surgical debridement.

**Case Series** As a Dedicated COVID Center (DCH), the institute received the largest population of COVID-19 mucormycosis patients from the entire eastern region of the country between May 2021 and August 2021. More than 5,000 COVID-19 were admitted out of which 218 patients were diagnosed with mucormycosis. Nine patients, seven males and two females, with a mean age of 39 years with craniofacial mucormycosis underwent debridement and early reconstructions (2–4 weeks from first debridement and start of antifungal therapy) with free and pedicled flaps. All flaps survived and showed no evidence of recurrence. The average time of the early reconstruction after surgical debridement was 1.7 weeks once the course of systemic amphotericin B was received.

**Conclusion** After aggressive surgical resection and a short course of antifungal therapy, early reconstruction can be done safely based on clinical criteria, as long as there is no evidence of hyphae invasion on wound edges in the intraoperative pathology examination.

The pandemic caused by the novel severe acute respiratory syndrome coronavirus 2 (SARS-CoV-2) has challenged health care professionals worldwide and continues to do so to date. In spite of all the efforts, the definitive treatment of coronavirus disease 2019 (COVID-19) remains controversial. Systemic steroid is one of the treatment modalities, but its unchecked use has adversely resulted in secondary bacterial and fungal infections. 1 Preexisting comorbidities such as diabetes mellitus, malignancy, immunocompromised states, lung diseases, etc. may also give rise to increased incidence of secondary infections. Secondary fungal infections are very rare, accounting for less than 1%, 2 but recent reports suggest an acute rise, especially in India. The risk of infection with *Aspergillus* , *Candida* , and *Pneumocystis jirovecii* in COVID-19 is well recognized, 3 4 5 6 but the unprecedented surge in mucormycosis has posed a new challenge. This uncommon but potentially fatal fungal infection first involves the nasal cavity and paranasal sinuses, which mimic the features of acute sinusitis, but in an immunocompromised individual, it has a tendency for rapid spread to orbit and intracranial sites. 7 8 9

Mucormycosis is essentially a medical disease, but surgical debridement of necrotic tissues is of paramount importance to increase the absorption and permeability of systemic antifungal medications. The resection includes almost all facial structures such as skin and muscle, nasal structure, paranasal sinuses, necrotic tissues in temporal and infratemporal fossa, and orbital exenteration. 10 The extensive debridement creates a variety of complex defects for reconstruction which pose a challenge to the plastic surgical team.

In this case series, we present our experience with the feasibility of early reconstruction after surgical debridement based on clinical criteria and the absence of hyphae invasion on wound edges in the intraoperative pathology examination.

## Management of Mucormycosis

**Fig. 1:**
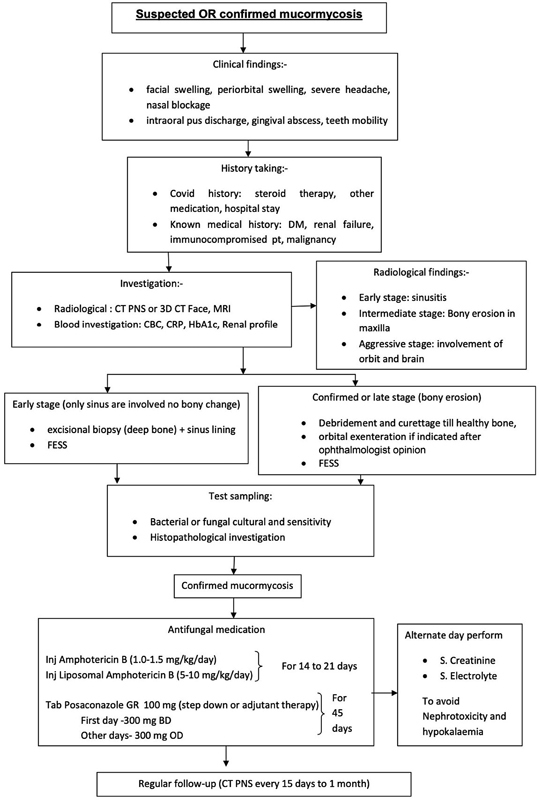
Institute standard-operating protocol for managing mucormycosis.

A management protocol for rhino-orbito-cerebral mucormycosis (ROCM) with craniofacial involvement was formulated at our institute ( Fig. 1 ). A multidisciplinary team consisting of ENT surgeons, neurosurgeons, ophthalmologists, and plastic surgeons was constituted. All the patients coming to the hospital with suspected mucormycosis were evaluated in special “mucor OPD”' and potassium hydroxide (KOH) mount from the nose and gadolinium-enhanced magnetic resonance imaging (MRI) of the nose, paranasal sinuses, orbit, and brain were advised. The diagnosis was mainly clinical–radiological based on history, nasal endoscopic findings of “blackish turbinate”/eschar/necrosis, contrast-enhanced MRI, and a positive KOH mount. Antifungal therapy started with intravenous liposomal amphotericin B (L-AmB) 5 to 10 mg/kg/d for 2 to 3 weeks. The step-down therapy was posaconazole 300 mg BD on day 1 followed by 300 mg OD for the next 45 days. Follow-up protocol included regular erythrocyte sedimentation rate and C-reactive protein (CRP) monitoring and signs of inflammation.

## Timing of Reconstruction

Due to the paucity of literature on reconstruction in post-COVID-19 mucormycosis defects, there was apprehension regarding the immediate reconstruction due to the aggressive nature of the disease, so the plastic surgery team at our center agreed upon selecting a time to maintain the balance between the disease aggression and prevention of tissue contractures and established deformities. We classified the timing of reconstruction after initial debridement as immediate—at the time of first surgical debridement, early—within 2 to 4 weeks of surgical debridement, and late—more than 4 weeks of surgical debridement. Early reconstruction was planned for all those who did not show any signs of further progression after initial debridement for a minimum of 2 weeks. The time of initial debridement coincided with the start of antifungal therapy. Late reconstruction was considered for patients who required frequent debridement due to the aggressiveness of the disease, visible both clinically and on biochemical parameters. They were monitored according to the management protocol of ROCM with continued antifungal therapy and underwent reconstruction only when no signs of further progress were there. The authors did not perform any immediate reconstruction.

## Surgical Protocol

**Fig. 2:**
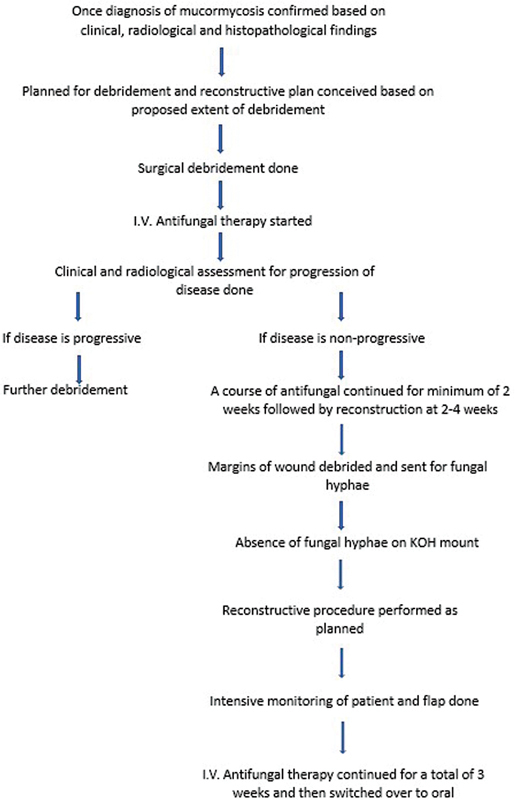
Reconstructive protocol for postdebridement mucormycosis defect.

Once the diagnosis was confirmed, a multispecialty team of surgeons evaluated the extent of resection using computed tomography (CT) and MRI films, and open surgical debridement was done followed by the daily dressing of the wound and intravenous L-AmB in the dose of 5 to 10 mg/kg/d was started. The disease progression was assessed clinically by signs of any visible inflammation and daily monitoring of biochemical parameters (blood sugar, CRP). Once a course of 2 weeks of intravenous amphotericin B was completed and no further progression of disease was confirmed, the patients underwent final debridement and reconstruction in the same sitting with a two-team approach as per reconstructive protocol ( Fig. 2 ). The volume and size of the anticipated defect to be reconstructed were calculated so as to choose an appropriate flap for reconstruction. To ensure that disease-free margins were achieved macroscopically, all affected tissues were resected up to a 1-cm margin of healthy tissue, and complete resection of involved bones was also attempted in case any remaining necrosis. When margins showed fresh bleeding and no affected tissue could be apparently seen, the flap harvest was started. In cases of free flaps, 1 to 2 mm of tissue from the recipient vessels, both artery and vein were sent for intraoperative hyphae analysis. All the other resected tissues were also sent to the pathology laboratory for KOH mount and hyphae analysis. Final histopathological reporting of the specimens was done after staining with methenamine silver. In the immediate postoperative period, patients were monitored in the intensive care unit and if the patient's response to the course of L-AmB was satisfactory, the systemic infusion was replaced by an oral tablet posaconazole in the dose of 300 mg BD on day 1 followed by 300 mg OD for the next 45 days.

## Results

During the pandemic, the institute was declared a Dedicated COVID Center (DCH) and received the largest population of COVID-19 patients and almost all post-COVID-19 mucormycosis patients from the whole state between mid-May 2021 and August 2021. More than 5,000 COVID-19 were admitted out of which 218 patients were diagnosed with mucormycosis. A total of 145 patients required surgical debridement, out of which 9 patients, 7 males, and 2 females, with a mean age of 43 years underwent early reconstruction with free and pedicled flaps for variable degrees of postdebridement defects ( Table 1 ). All of them were treated with intravenous amphotericin B and reconstruction was done within 2 to 4 weeks of debridement in all cases. The patients who had late reconstructions (>4 weeks) were excluded from this series.

**Table 1:** Case description of craniofacial defects in post-COVID-19 mucormycosis

Case	Sex/age	Past history of COVID-19	Comorbidities	Surgical resection	Reconstruction	Outcome	Follow-up duration (in mo)
1	F/38	+	DM2	Right hemifacial skin resection + subtotal maxillectomy (type 2)	Reconstructed with a free radial artery forearm flap	Flap survival and asymptomatic at 2 mo follow-up	8
2	M/47	+	DM2	Left orbital exenteration and frontal skin excision	Reconstructed with temporalis muscle with cheek advancement flap	Flap survived and the patient was asymptomatic	10
3	M/56	−	DM2	Right segmental mandibulectomy with resection of submandibular gland	Mandibular stabilization with plate followed by reconstruction with free fibula	Patient was asymptomatic	20
4	F/52	+	DM2	Left hemifacial skin resection + limited maxillectomy (type 1)	Forehead flap for wound dehiscence of cheek advancement flap	Flap survived and the patient was asymptomatic	6
5	M/48	+	DM2	Excision of left half of palate	Palate reconstruction with temporalis muscle	Flap survived and the patient was asymptomatic	9
6	F/42	+	None	Right hemifacial skin resection + subtotal maxillectomy (type 2)	Reconstructed with a free radial artery forearm flap	Flap survival and asymptomatic at 2 months follow-up	6
7	M/50	+	DM2	Excision of left half of palate	Palate reconstruction with temporalis muscle	Flap survived, but the patient expired due to a chest infection	NA
8	M/48	+	DM2	Right orbital exenteration and frontal skin excision	Reconstructed with temporalis muscle with cheek advancement flap	Flap survived and the patient was asymptomatic	10
9	M/40	+	None	Left orbital exenteration and frontal skin excision	Reconstructed with temporalis muscle with cheek advancement flap	Flap survived and the patient was asymptomatic	6

Abbreviations: COVID-19, coronavirus disease 2019; DM2, diabetes mellitus type 2; NA, not available.

Two patients underwent free radial artery forearm flap for cavity filling and skin coverage. In three patients, the orbital defect was covered with the temporalis muscle for cavity filling and advancing cheek flaps. In two patients, the palatal defect was reconstructed with a temporalis muscle flap. One patient underwent a forehead flap for the residual defect in the skin after the medial dehiscence of the cheek advancement flap. One patient with mandible involvement underwent segmental mandibulectomy and stabilization with a mandibular plate in the first stage and reconstruction with a free fibula osseous flap in the second stage. A major reason for circumventing the use of free flaps in most of these patients was the patients' and family members' refusal to undergo microsurgical reconstructions. As the second wave of the pandemic was ongoing, they just wanted to get their lives saved from COVID-19 and mucormycosis with minimum interventions and to get discharged from the hospital at the earliest.

In all cases, no flap or wound healing-related complication was encountered, although one patient (case 7) died on day 10 and there was a dehiscence of cheek advancement flap in one case for which secondary reconstruction was done (case 4). None of the patients showed recurrence of the fungal infection, flap loss, or any adverse effect related to cerebral invasion. In this series, the flaps used for reconstruction provided good coverage for the defects with acceptable esthetic results in the patients. The mean follow-up period was 9.3 ± 4.6 months. We are mentioning the case details of only specific reconstruction that was done for these patients.

## Case Series

### Case 1

**Fig. 3:**
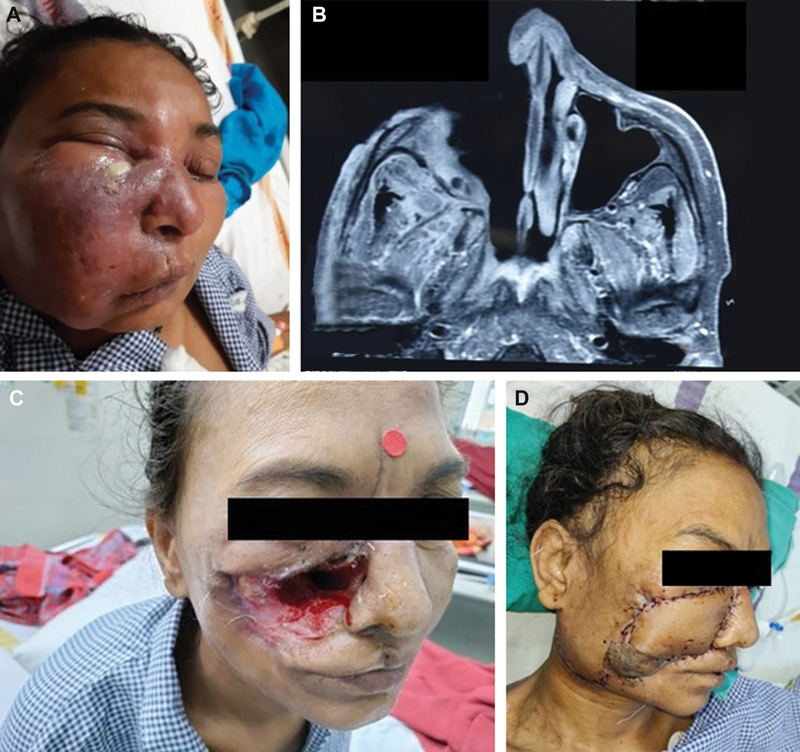
(A) Clinical picture of mucormycosis over right cheek. (B) Magnetic resonance imaging showing the defect in the maxilla. (C) Midface defect after debridement. (D) Defect reconstructed with free radial artery forearm flap.

A 55-year-old woman presented with complaints of right-sided facial swelling, intense pain radiating to her right eye, and inability to open the ipsilateral eye for the last 2 weeks. There was associated numbness and pus discharge from the swollen area. The patient was already reverse transcription polymerase chain reaction positive and received supportive medications for COVID-19 including steroids for 2 weeks and on oxygen support. She then underwent an MRI of the brain and paranasal sinus which showed features of sinusitis in the right maxillary and paranasal sinuses, extensive involvement of the nose, and some part of the orbital floor. She was referred to the institute where an endoscopic biopsy confirmed the presence of mucormycosis. An open surgical debridement was done followed by daily dressing of the wound and intravenous L-AmB 5 to 10 mg/kg/d was started. After a course of 2 weeks of antifungal therapy, she underwent final debridement, and the midface defect was reconstructed with a free radial artery forearm flap forming both the medial (lining) and anterior (cover) walls of the maxilla ( Fig. 3A–D ).

### Case 2

**Fig. 4:**
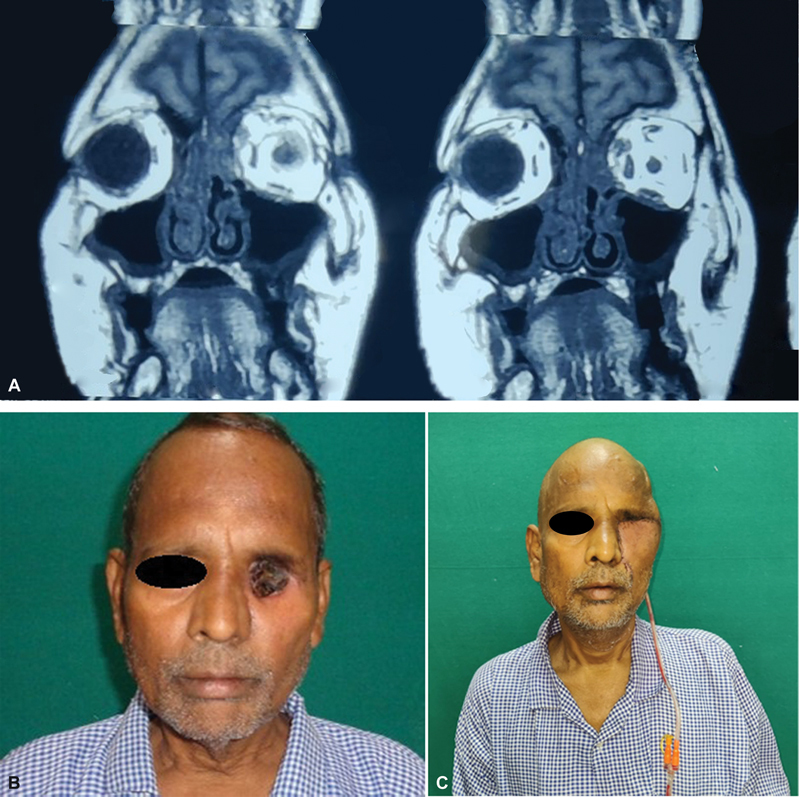
(A) Magnetic resonance imaging showing left orbital involvement. (B) Postdebridement defect. (C) Reconstruction with temporalis muscle and cheek advancement flap.

A 47-year-old man developed recurrent high-grade fever, left-sided headache, and pain in the left eye radiating to the face with difficulty in opening his left eye and poor vision approximately 1 and a half weeks back. On MRI, extensive involvement of the orbit was found, and biopsy revealed hyphae, so targeted systemic therapy in the form of intravenous L-AmB 5 to 10 mg/kg/d, was started. He underwent debridement with left orbital exenteration and left limited maxillectomy with primary closure of facial skin. A week following, there was pain, redness, and gaping of the suture line for which daily dressing of the wound was done. Once the pus discharge decreased, the patient underwent temporalis muscle to fill the orbital cavity, and a cheek advancement flap was used for skin lining ( Fig. 4A–C ).

### Case 3

**Fig. 5:**
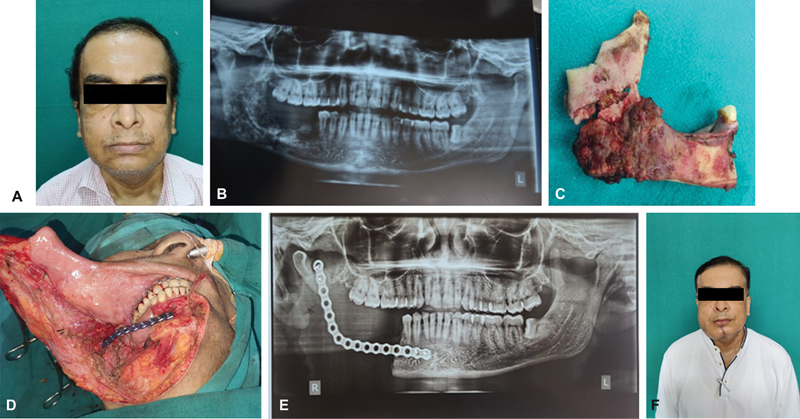
(A) Preoperative photograph with mild swelling evident over left lower jaw. (B) Orthopantomogram showing honeycomb appearance and osteomyelitis of the right mandible. (C) Blackish discoloration of the bone with thinned-out cortex at the junction of body and ramus. (D) Defect after segmental mandibulectomy and reconstruction plate in situ. (E) Orthopantomogram showing mandible plate in situ. (F) One month follow-up after mandible reconstruction with free fibula osseous flap.

A 56-year-old man presented with pain and swelling on the right side of the lower jaw for 1 week following COVID-19-positive status. Orthopantomogram showed the honeycomb appearance of the body and ramus of the right mandible along with features of osteomyelitis of both the outer and inner cortices. MRI also confirmed the involvement of the right side of the mandible. Segmental mandibulectomy and stabilization with a reconstruction plate followed by mandible reconstruction at a later stage were discussed with the patient and family members. Intraoperatively, the body, ramus, and coronoid process on the right side were removed and black fungal colonies could be seen in between the cortices. Intravenous L-AmB 5 to 10 mg/kg/d was started along with monitoring of potassium and sugar levels. There was some discharge from the wound site for 5 to 7 days. After 2 weeks of intravenous therapy, he was started on oral posaconazole tablet as 300 mg BD on day 1 followed by 300 mg OD, and mandibular reconstruction with a free fibula osseous flap was done once there were no further signs of disease progression ( Fig. 5A–F ).

### Case 4

**Fig. 6:**
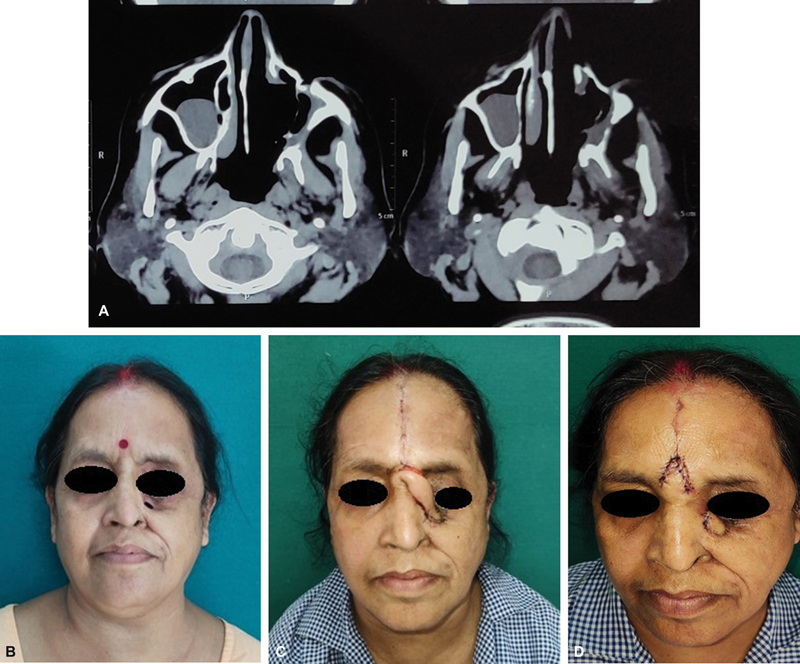
(A) Postdebridement defect in left maxilla. (B). Defect after dehiscence of cheek advancement flap. (C) Median forehead flap in situ. (D) Flap after division and insetting.

A 52-year-old woman had a low-grade fever, left-sided facial swelling, pain and numbness, blood-tinged discharge from her left nose, and inability to open her left eye for a duration of 2 weeks. At the institute, she was clinically diagnosed with nasal mucormycosis with COVID-19-positive status, so open surgical debridement and removal of sequestrated bone with cheek advancement were done. Intravenous L-AmB in the dose of 5 to 10 mg/kg/d was started and the patient showed signs of improvement. But subsequently, the medial edge of the suture line was dehisced for which a median forehead flap was done as the patient and attendants requested for minimal reconstructive procedure with acceptance of some deformity. Two weeks later, the flap was divided, and the patient did well without any recurrence of mucormycosis ( Fig. 6A–D ).

### Case 5

**Fig. 7:**
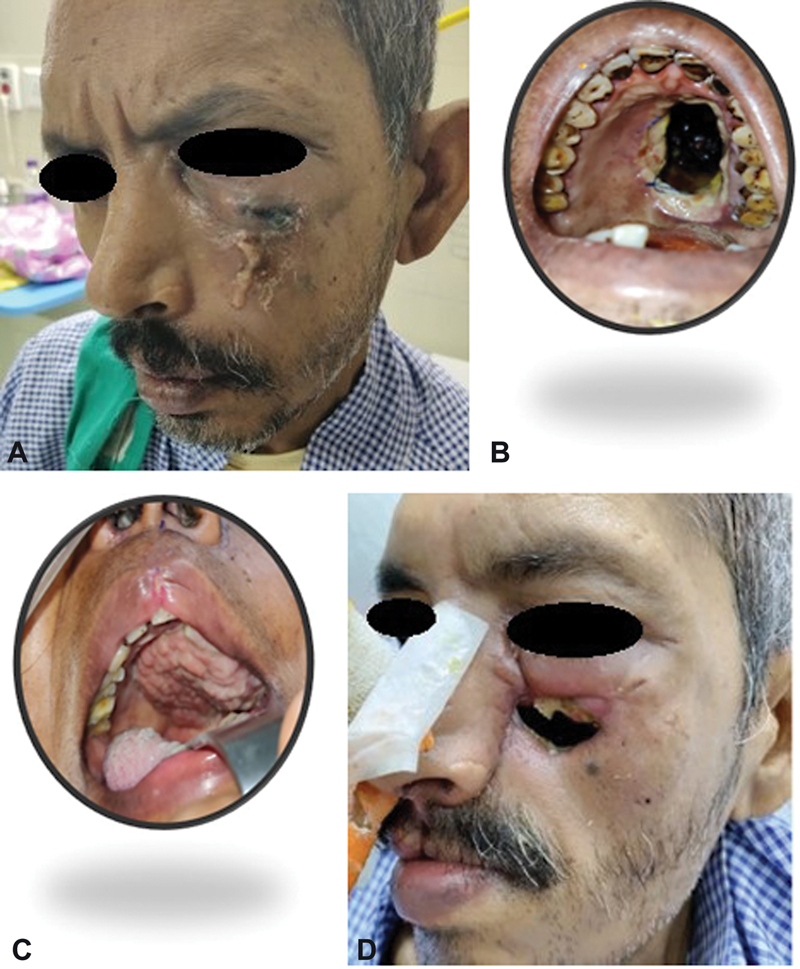
(A) Involvement of left maxilla and skin. (B) Defect in left palate after debridement. (C) Palate reconstructed with temporalis muscle. (D) Defect after dehiscence of cheek advancement flap.

A 48-year-old man complained of left facial pain and high-grade fever for a week followed by redness and pus discharge over the infraorbital region. He also noticed a black patch over his palate which gradually increased in size. He was not a known case of diabetes, but his blood sugar at the time of admission was very high (>350 mg/dL) and uncontrolled on medications. KOH sample from the skin showed fungal hyphae for which debridement was done, and intravenous L-AmB 5 to 10 mg/kg/d was given for 2 weeks. Postdebridement CT of the face showed defects of the anterior wall of the maxilla and also the inferior wall (left palate). The patient underwent temporalis muscle for palate and lining reconstruction and a cheek advancement flap for skin cover. Later, there was a dehiscence of the suture line near the medial canthus and the patient underwent a forehead flap ( Fig. 7A–C ).

## Discussion

With the insurgence of the COVID-19 pandemic, the cases of mucormycosis reported in COVID-19 patients have been the highest in India with a prevalence of 0.14 per 1,000 which is nearly 80 times higher compared with developed countries. 11 12 13 14 Another implicated cofactor in the resurgence of the disease may be attributed to environmental factors such as the presence of warm temperatures (> 25°C) and humidity (≥70%). 15

The disease spreads from the nasal or the oral cavity to the adjacent structures such as the paranasal sinus, orbit, and intracranial region as these structures are separated from the nasal cavities by only thin bones (lateral lamella and cribriform plate). 16 17 The pathogenesis of craniofacial mucormycosis is due to the thrombiforming property of the invasive fungus leading to occlusion of the blood vessels and subsequent ischemia and necrosis of the tissues. 15 The peripheral microangiopathy caused due to diabetes mellitus and the prolonged use of steroids might exaggerate the process. 13 14 15 16 18 Another factor that can be considered in the spread of the disease is a history of previous surgery such as tooth extraction. Two of our patients had a prior history of tooth extraction which may cause the direct spread of the disease in the bloodstream. In our study, seven out of nine patients (88.9%) had diabetes mellitus with five of them having an HbA1c more than 10%. This is in accordance with Kumari et al who have also reported that 80% of their reconstructed patients suffer from diabetes mellitus. 19

The mainstay of treatment of craniofacial mucormycosis is phasic debridement, systemic antifungal therapy, and reconstruction of the involved structures. The protocol for antifungal therapy followed at our institute was intravenous L-AmB in a dose of 5 mg/kg body weight. The total dose is then diluted in 5% dextrose considering that each vial contains 50 mg of amphotericin B. In case of central nervous system involvement, the dose is increased to 10 mg/kg body weight. This treatment is usually continued for 2 to 3 weeks till the acute disease is stabilized along with serial debridement of necrosed tissue. After a period of 2 to 3 weeks of L-AmB therapy, it is shifted to oral posaconazole 300 mg delayed-released tablets twice a day for 1 day followed by 300 mg daily for 1.5 months. In our study, the mean duration of intravenous amphotericin B therapy was 15 days. The average number of debridements required before the flap reconstruction ranged from 1 to 4 (mean—2.4) and the mean duration till completion of surgical debridement was 1.7 weeks. Of the total 218 patients admitted to our center for mucormycosis, there were 41 deaths (18.8%). The mortality rate in mucormycosis is fairly high in the range of 36 to 44%. 20 These cases therefore require a high level of vigilance, prompt diagnosis, and management.

Mucormycosis unforgivingly involves all of the tissues in the local area right from the skin to the bones. Debridement of these necrotic tissues leaves variable defects ranging from small skin defects which could be primarily closed to extensive defects of cheek skin, mandibular and hemipalatal defects, and even large cavities extending to involve orbital exenteration defects. Reconstructing such defects remains a challenge for reconstructive surgeons not only in relation to the magnitude of the defects but also in relation to the ongoing disease process. Reconstruction in such patients is urgently required as bones exposed after debridement for a long duration may lead to desiccation and necrosis of bones. But at the same time, eradication of the disease has to be confirmed which otherwise may perpetuate the infection leading to masking of the infection and fulminant spread associated with flap invasion or flap loss. Such consequences impact the morbidity and even mortality in such patients. The most ideal way of reconstructing any defects is “like by like” which can only be followed to a limited extent considering the associated comorbidities in such patients. The primary goal of reconstruction in all our patients was to restore functions in the form of deglutition and speech to the maximum extent possible. The choice of procedures done in our cases depended on the excised components and extent of the defect as well as patients' preference. It could be argued that the blood supply is precarious in post-COVID-19 mucormycosis patients both due to the presence of a hypercoagulable state as well as the direct local involvement of vessels; however, all flaps in our series survived with no skin complications except dehiscence in one case. Coverage of such large defects could undeniably lead to many complications. The various complications that can occur are oronasal fistula, 21 22 naso-orbital fistula, 23 24 and oropharyngeal fistula. 25 Various other complications such as exposed meninges, 21 cerebrospinal fluid leak, 26 and even temporomandibular joint ankylosis 27 have also been reported. In our series of nine patients, none of these complications occurred. Flap survived in all patients with good wound healing and acceptable esthetic and functional results.

## Conclusion

The unprecedented surge of mucormycosis in post-COVID-19-infected patients particularly in our part of the Asian subcontinent is an emerging challenge. The highly invasive nature of this disease warrants prompt diagnosis and a combination of aggressive medical and surgical management. Both pedicled and free flaps after thorough serial debridement with concomitant use of intravenous antifungal therapy are the mainstay of management. An early reconstruction is acceptable with advantages while accepting minor surgical complications such as wound dehiscence. So, a balance between the aggressive nature of mucormycosis infection and successful reconstructive procedures is the key element for good functional and esthetic outcomes.
